# Effects of drug pressure and human migration on antimalarial resistance in circulating Plasmodium falciparum malaria parasites in Ecuador

**DOI:** 10.21203/rs.3.rs-4638168/v1

**Published:** 2024-08-16

**Authors:** Isaac Ñacata, Angela M. Early, Janeth Boboy, Daniel E. Neafsey, Fabián E. Sáenz

## Abstract

Antimalarial resistance in *Plasmodium falciparum* is a public health problem in the fight against malaria in Ecuador. Characterizing the molecular epidemiology of drug resistance genes helps to understand the emergence and spread of resistant parasites. In this study, the effects of drug pressure and human migration on antimalarial resistance in *P. falciparum* were evaluated. Sixty-seven samples from northwestern Ecuador from the 2019–2021 period were analyzed. SNPs in *Pfcrt* , *Pfdhps* , *Pfdhfr* , *Pfmdr-1* , *Pfk13* and *Pfaat1* were identified by Sanger sequencing and whole-genome sequencing. A comparison of the frequencies of the haplotypes was made with data from the 2013–2015 period. Also, nucleotide and haplotype diversity were calculated. The frequencies of the mutant haplotypes, CVM **ET** in *Pfcrt* and C **I** C **N** I in *Pfdhfr* , increased. NED **F** S **D** F **Y** in *Pfmdr-1* was detected for the first time. While the wild-type haplotypes, SAKAA in *Pfdhps* and MYRIC in *Pfk13* , remained dominant. Interestingly, the A16 **V** mutation in *Pfdhfr* that gives resistance to proguanil is reported in Ecuador. In conclusion, parasites resistant to chloroquine ( *Pfcrt* ) and pyrimethamine ( *Pfdhfr* ) increased in recent years, while parasites sensitive to sulfadoxine ( *Pfdhps* ) and artemisinin ( *Pfk13* ) prevail in Ecuador. Therefore, the current treatment is still useful against *P. falciparum* . The frequent human migration between Ecuador and Colombia has likely contributed to the spread of resistant parasites. **Keys words** : *Plasmodium falciparum* , resistance, antimalarial, selective pressure, human migration.

## Introduction

Malaria is a vector-borne infectious disease that is endemic in tropical and subtropical regions of the world [1]. *Plasmodium falciparum* causes the most severe malaria in humans and the highest morbidity and mortality worldwide [2]. According to the World Health Organization (WHO), in 2022, 249 million cases and 608 000 deaths by malaria occurred in the world, and 600 000 cases and an incidence of 3.6 cases per 1000 inhabitants occurred in the Americas [3]. In this region, the highest malaria incidence is attributed to *P. vivax* followed by *P. falciparum* [1,4].

Since the approval of artemisinin-based combination therapy (ACT) against *P. falciparum*, several South American countries have implemented it in their treatment schemes [5,6]. Artemether (AR) and artesunate (AS) are the most common artemisinins (ARTs) used in ACTs [5]. Currently, seven of eleven countries in this region, including Ecuador, use artemether-lumefantrine (AL) + primaquine (PQ) as first-line treatment and quinine (QN) + clindamycin (CD) as second-line treatment [5,7].

Antimalarial resistance in *P. falciparum* is a public health problem that complicates malaria control in endemic regions [8,9]. Resistance is an evolutionary response to pressure exerted by drugs over parasites that allows them to survive and reproduce (fitness) during exposure to antimalarials [6]. This advantage is due to mutations that modify protein function and reduce antimalarial efficacy [10,11]. In *P. falciparum*, Single Nucleotide Polymorphisms (SNPs) have been identified in specific genes that are related to resistance to antimalarials such as chloroquine (CQ), sulfadoxine-pyrimethamine (SP), and ARTs, among others [12–14].

CQ resistance in *P. falciparum* originated in Southeast Asia in the late 1950s [14]. Worldwide, *P. falciparum* exhibits CQ resistance mediated by mutations in *Pfcrt* that change the configuration of the chloroquine resistance transporter (PfCRT), located in the digestive vacuole (DV), allowing drug expulsion [15,16]. High concentrations of CQ in the DV prevent the release of toxic endogenous products, which cause the death of the parasite [17]. Mutations at codons 72–76 are associated with CQ resistance [18,19]. *In vitro* studies show that all chloroquine-resistant parasites have the K76**T** mutation, essential to confer CQ resistance [15,19]. In the early 1960s, CQ resistance emerged independently in South America [14], and CVMN**T**, CVM**ET** and CV**EIT** haplotypes have been found to predominate on the Pacific coast and **S**VMN**T** in the Amazon region [20,21]. Specifically, in Ecuador, CVMN**T**, CVM**ET** and **S**VMN**T** have been previously reported [20,22]. In addition, other mutations such as H97**Q**, A220**S**, Q271**E**, N326**D**, C350**R**, I356T/**L** and R371**I** in *Pfcrt* contribute to different degrees of CQ resistance [15,23,24]. In South America, H97**Q**, A220**S**, N326**D**, S334**N**, I356**L** and R371**I**/**T** have been found [16,17,23,25]. While in Ecuador, A220**S**, N326**D** and I356**L** with CVMN**T** were identified in Ecu1110, a CQ resistant isolate from 1990 [16,20].

Resistance to the SP drug combination in *P. falciparum* originated in Southeast Asia in the 1960s [14]. In South America, SP treatment failure has been registered since the 1980s [26]. SP resistance is associated with mutations in *Pfdhps* and *Pfdhfr*, which encode synergistic enzymes: dihydropteroate synthetase (DHPS) and dihydrofolate reductase (DHFR), respectively [13,27]. Both enzymes participate in the folate synthetic pathway, essential for DNA synthesis and the metabolism of some amino acids (29). The presence of SP causes competitive inhibition of the active site of DHPS and DHFR, causing parasite death [28,29]. Mutations at codons 436, 437, 540, 581 and 613 in *Pfdhps* confer sulfadoxine resistance. In South America, SAKAA (wild type), **F**AKAA, **FG**KAA, S**G**KAA, SA**E**AA, S**G**K**G**A and S**GEG**A have been reported (Corredor et al., 2010; Cortese et al., 2002b; Fierro, 2017; Griffing et al., 2011; Mita et al., 2011), while only SAKAA and SA**E**AA have been found in Ecuador [20]. On the other hand, mutations at codons 50, 51, 59, 108 and 164 in *Pfdhfr* confer pyrimethamine resistance [34]. *In vitro* studies show that S108**N** is the first mutation to emerge in *Pfdhfr*, which is essential to cause pyrimethamine resistance [34,35]. In South America, CNCSI (wild-type), CNC**N**I, C**I**C**N**I, C**I**C**NL**, CNC**T**I and **RI**C**N**I haplotypes have been reported (Corredor et al., 2010; Cortese et al., 2002b; Fierro, 2017), while CNCSI, CNC**N**I and C**I**C**N**I have been found in Ecuador [20].

Mutations in *Pfmdr-1* as well as its copy number are associated with resistance to several drugs such as CQ, QN, amodiaquine (AQ), mefloquine (MQ) and lumefantrine (LF) [15,36]. Mutations at codons 86, 130, 144, 184, 1034, 1042, 1226 and 1246 change the charge and structure of the multidrug resistance transporter (PfMDR1), located in the DV, causing drug expulsion [37,38]. N86**Y**, Y184**F**, S1034**C**, N1042**D** and D1246**Y** mutations have been reported in South America [24,36], while Y184**F** and N1042**D** have been found in Ecuador [20].

ART resistance in *P. falciparum* was first reported in Southeast Asia in 2006 [39]. Initially, ARTs are activated by heme and iron to then cause protein and proteasome damage, leading to prolonged cellular stress and parasite death [40]. Mutations at codons 476, 493, 539, 543 and 580 in *Pfk13* are associated with ART resistance [36,40,41]. In South America, C580**Y** has only been found in Guyana since 2010 [41,42], while Ecuador, Colombia, Peru, Venezuela, Brazil, Suriname and French Guiana have not reported any mutation associated with ART resistance [20,42,43].

In recent years, amino acid transporter 1 (*Pfaat1*) has been associated with resistance to quinolines such as QN, AQ MQ and especially CQ [44–46]. A study suggests that the PfAAT1 protein, located in the DV, transports amino acids out of the DV while allowing entry of quinolines [46,47]. However, the presence of mutations such as T162**E** changes the charge of PfAAT1, blocking drug entry into the DV, and causes resistance to CQ, QN, AQ and MQ [46]. One PfAAT1 mutation with demonstrated CQ-resistance phenotype (S258**L**) was found on the Pacific coast of Ecuador and Colombia [48,49].

In South America, *P. falciparum* populations exhibit varying degrees of enhanced relatedness as a result of bottlenecks caused by efforts to eliminate malaria in the region [11,21,50]. In Esmeraldas, Ecuador, a malaria outbreak was caused by a clonal expansion in 2013 [21]. In addition, malaria outbreaks are influenced by the spread of parasites through human hosts between endemic regions [21,22,51]. In the malaria-endemic countries of the Americas, 7775 imported cases were reported in 2016 and 8411 in 2017, while in Ecuador, the latest records reported 395 cases between 2015 and 2017, with 41.5% of them were due to *P. falciparum* [5,52].

In 2012, Ecuador was considered one of 21 countries in the world with the potential to eliminate malaria by 2020 due to a reduction in cases since 2002 of more than 99% [2]. The decrease in malaria infections was largely due to the success of the malaria elimination programs; however, infections have increased since 2015 [4]. This increase may be associated with factors such as the restructuring of the health system, climate change, indiscriminate drug use and the spread of parasites by vector and host [53,54]. Genetic surveillance can help to resolve whether the emergence of new resistant genotypes in *P. falciparum* are the result of de-novo mutations or the spread of parasites by human host migration.

This study focuses on the molecular epidemiology of drug resistance genes to determine the effect of drug pressure and human migration on antimalarial resistance in *P. falciparum.* This work aims to characterize the current epidemiological situation of resistance, contribute to the strengthening of health programs and notify the efficiency of the treatment schemes against *P. falciparum* in Northwest Ecuador (Fig. 1).

### Results

## Resistance genotypes (2019–2021)

Sixty-seven *P. falciparum* samples were analyzed by Sanger sequencing of the genes: *Pfcrt* (codons 72–76), *Pfdhps* (codons 436, 437, 540, 581 and 613), *Pfdhfr* (codons 50, 51, 59, 108 and 164), *Pfmdr-1* (codons 86, 130, 144, 184, 1034, 1042, 1226 and 1246) and *Pfk13* (codons 476, 493, 539, 543 and 580) to identify SNPs and determine drug resistance genotypes. Samples of local Ecuadorian cases corresponded to the counties of Esmeraldas (n = 27), San Lorenzo (n = 5) and Tobar Donoso (n = 18). Samples obtained from Colombia (n = 17) corresponded to migrants from Nariño, Colombia who were diagnosed in San Lorenzo, Ecuador.

The PfCRT haplotypes found in 63 samples were: 61.90% CVM**ET** (39/63), 36.51% CVMN**T** (23/63) and 1.59% CV**IET** (1/63). CVM**ET** was dominant in Esmeraldas (25/25). In San Lorenzo, the CVM**ET** proportion (3/5) was higher than CVMN**T** (2/5). In Tobar Donoso, CVMN**T** had the higher proportion (16/18), followed by CVM**ET** (2/18). In samples obtained from Colombia, CVM**ET** (9/15) was the most prevalent haplotype followed by CVMN**T** (5/15) and CV**IET** (1/15) (Fig. 2a). The CVM**ET**, CVMN**T** and CV**IET** haplotypes contain the M74**I**, N75**E** and K76**T** mutations associated with CQ resistance [23].

The PfDHPS haplotypes found in 52 samples were: 98.08% the wild-type SAKAA (51/52) and 1.92% S**G**KAA (1/52), a potentially resistant haplotype. SAKAA was dominant in Esmeraldas (24/24), San Lorenzo (5/5) and Tobar Donoso (17/17). While in samples obtained from Colombia, SAKAA was most prevalent (5/6) followed by S**G**KAA (1/6) (Fig. 2b). In addition, a synonymous mutation (AA**G**) at codon 540 was found in a sample from Esmeraldas and Tobar Donoso and two samples from Colombia. The S**G**KAA haplotype contains the A437**G** mutation associated with resistance to sulfadoxine [32].

The PfDHFR haplotypes found in 63 samples were: 63.49% C**I**C**N**I (40/63), a resistant haplotype, the wild-type 34.92% CNCSI (22/63), and 1.59% CNC**N**I (1/63), a potentially resistant haplotype. C**I**C**N**I was dominant in Esmeraldas (27/27). In San Lorenzo, the C**I**C**N**I proportion (3/5) was higher than CNCSI (2/5). All Tobar Donoso samples presented the wild-type, CNCSI (18/18). In samples obtained from Colombia, C**I**C**N**I (10/13) was the most prevalent followed by CNCSI (2/13) and CNC**N**I (1/13) (Fig. 2c). The C**I**C**N**I and CNC**N**I haplotypes contain the N51**I** and S108**N** mutations associated with pyrimethamine resistance [29].

The PfMDR-1 haplotypes found in 58 samples were: 53.45% NED**F**S**D**FD (31/58) and 46.55% NED**F**S**D**F**Y** (27/58). NED**F**S**D**FD proportion was higher in Tobar Donoso (18/18) than Esmeraldas (3/23), while NED**F**S**D**F**Y** was predominant in Esmeraldas (20/23). In San Lorenzo, NED**F**S**D**FD (3/5) was the most prevalent followed by NED**F**S**D**F**Y** (2/5). In samples obtained from Colombia, the NED**F**S**D**FD proportion (7/12) was higher than NED**F**S**D**F**Y** (5/12) (Fig. 2d).

The PfK13 haplotype found in 52 samples was: 100% the wild-type MYRIC (52/52). MYRIC was present in all samples from Esmeraldas (17/17), San Lorenzo (5/5), Tobar Donoso (18/18) and samples obtained from Colombia (12/12) (Fig. 2e).

### Whole-genome sequencing (2019–2021)

*P. falciparum* samples underwent whole-genome sequencing (WGS) following selective whole genome amplification (SWGA) to boost representation of *P. falciparum* DNA within total DNA extracts [55]. WGS could be performed on fifty-four of the sixty-seven *P. falciparum* samples.

WGS coverage varied across sites and samples but confirmed the mutations found by Sanger sequencing in *Pfcrt* (codons 72–76) in 36 samples, *Pfdhps* (codons 436, 437, 540, 581 and 613) in 37 samples, *Pfdhfr* (codons 50, 51, 59, 108 and 164) in 35 samples and *Pfmdr-1* (codons 86, 130, 144, 184, 1034, 1042, 1226 and 1246) in 34 samples.

In addition, WGS found other mutations that were not identified by Sanger sequencing in *Pfcrt* (codons 97, 220, 326, 334, 356 and 371), *Pfdhps* (codon 142), *Pfmdr-1* (codon 35), *Pfk13* (codons 189 and 344) and *Pfaat1* (codons 80, 231 and 258). These mutations are detailed in Table 1.

Table 1
**Frequencies of SNPs found in drug resistance genes obtained by WGS**. n: Number of samples, SNP: Single nucleotide polymorphism.

| Gen | n | Percentage | SNP |
|---|---|---|---|
| *Pfcrt* | 19/36 | 52.78% | H97**Q** |
| | 36/36 | 100% | A220**S** |
| | 15/37 | 40.54% | N326**D** |
| | 15/37 | 40.54% | S334**N** |
| | 16/38 | 42.11% | I356**L** |
| | 21/38 | 55.26% | R371**T** |
| *Pfdhps* | 16/36 | 44.44% | K142**K**(AA**A**) |
| *Pfdhfr* | 2/30 | 6.67% | A16**V** |
| *Pfmdr-1* | 2/23 | 8.70% | I35**K** |
| *Pfk13* | 19/19 | 100% | K189**T** |
| | 2/19 | 10.53% | A344**V** |
| *Pfaat1* | 7/7 | 100% | D80**E** |
| | 19/33 | 57.58% | V231**D** |
| | 17/35 | 48.57% | S258**L** |

We found the A16**V** DHFR mutation in two samples from Tobar Donoso that have a clonal relationship (genome-wide identity by descent (IBD) > 0.99). This clone was not found within a set of 219 previously sequenced *P. falciparum* genomes that were sampled from the Pacific Coast from 1999 to 2017[49].

However, it does have high relatedness (IBD = 0.71) to clonal cluster G from Carrasquilla et al (2022)[49], which also carried the A16**V** DHFR variant. We examined the IBD tracts shared between these genomes and found that our new isolates and cluster G are IBD across a 0.8 Mb segment of chromosome 4, which includes the DHFR coding region. Genome wide, the extended length of the IBD segments, which in some instances cover entire chromosomes, suggests the genomes are separated by only a single recombination event. Given the temporal order of sampling, the Tobar Donoso genomes are likely the progeny of a cross between cluster G and another parasite, but we were unable to identify the second putative parent within our extended Pacific Coast data set.

## Comparative frequencies of drug resistance haplotypes over time

The spatial and temporal comparison allowed us to characterize the fluctuation of haplotype frequencies between the 2013–2015 and 2019–2021 periods in northwest Ecuador. In *Pfcrt*, CVM**ET** frequency increased in San Lorenzo (from 0.22 to 0.60; Chi-square test, n.s., significantly increased in Esmeraldas (from 0.03 to 1; Chi-square test, $P < 0.0001$) and appeared in Tobar Donoso (from 0 to 0.11; Chi-square test, n.s.). On the other hand, CVMN**T** frequency decreased in San Lorenzo (from 0.78 to 0.40; Chi-square test, n.s.), in Tobar Donoso (from 1 to 0.89; Chi-square test, n.s.) and disappeared in Esmeraldas (from 0.97 to 0; Chi-square test, $P < 0.0001$) (Fig. 3a). In *Pfdhps*, SAKAA frequency remained high in San Lorenzo, Esmeraldas and Tobar Donoso (Fig. 3b). In *Pfdhfr*, the wild-type CNCSI frequency significantly decreased in San Lorenzo (from 0.83 to 0.4; Chi-square test, $P = 0.003$) and disappeared in Esmeraldas (from 0.97 to 0; Chi-square test, $P < 0.0001$), but it remained high in Tobar Donoso (Chi-square test, n.s ). CNC**N**I disappeared in San Lorenzo (from 0.13 to 0; Chi-square test, n.s.), while C**I**C**N**I frequency significantly increased in Esmeraldas (from 0.03 to 1; Chi-square test, $P < 0.0001$) and San Lorenzo (from 0.04 to 0.60; Chi-square test, $P = 0.003$) (Fig. 3c). In *Pfmdr-1*, NED**F**S**D**FD frequency significantly decreased in Esmeraldas (from 0.97 to 0.13; Chi-square test, $P < 0.0001$) and San Lorenzo (from 0.78 to 0.60; Chi-square test, $P = 0.005$), but it remained high in Tobar Donoso (Chi-square test, n.s.). While NED**F**S**D**F**Y** appeared in Esmeraldas (from 0 to 0.87; Chi-square test, $P < 0.0001$) and San Lorenzo (from 0 to 0.4; Chi-square test, $P = 0.005$). Surprisingly, haplotypes with single mutations NED**F**SNFD (Y184**F**) and NEDYS**D**FD (N1042**D**) found in the 2013–2015 period disappeared in San Lorenzo (from 0.22 to 0) and Esmeraldas (from 0.03 to 0), although these changes were not statistically significant (Fig. 3d; Chi-square test, n.s.). Finally, in *Pfk13*, the wild-type MYRIC frequency has remained high in San Lorenzo, Esmeraldas and Tobar Donoso (Fig. 3e).

## Genetic diversity of drug resistance genes

The nucleotide diversity (π) and haplotype diversity ($H_d$) were calculated to determine the genetic diversity of *Pfcrt*, *Pfdhps*, *Pfdhfr* and *Pfmdr-1* in *P. falciparum* from northwest Ecuador and samples obtained from Colombia. The nucleotide diversity (π) measured the degree of DNA polymorphism, while haplotype diversity ($H_d$) measured the singularity of a haplotype in relation to sample size [56]. At *Pfcrt*, samples obtained from Colombia ($\pi = 0.080$, $H_d$=0.562) and San Lorenzo ($\pi = 0.080$, $H_d$=0.600) showed higher diversity than Tobar Donoso (π = 0.028, Hd = 0.209) and no diversity was found in Esmeraldas. At *Pfdhps*, samples obtained from Colombia (π = 0.080, $H_d$=0.600) had higher diversity than Tobar Donoso (π = 0.008, $H_d$=0.118) and Esmeraldas (π = 0.005, $H_d$=0.080) and no diversity was found in San Lorenzo. At *Pfdhfr*, San Lorenzo (π = 0.080, $H_d$=0.600) had higher diversity than Colombia (π = 0.040, $H_d$=0.410) and no diversity was found in Esmeraldas and Tobar Donoso. At *Pfmdr-1*, samples obtained from Colombia (π = 0.022, $H_d$=0.530) and San Lorenzo (π = 0.025, $H_d$=0.600) had higher diversity than Esmeraldas (π = 0.009, $H_d$=0.220) and no diversity was found in Tobar Donoso (Table 2). *Pfk13* was excluded from the analysis because it did not exhibit polymorphisms in the molecular markers analyzed.

Table 2

**Genetic diversity of molecular markers of drug resistance genes in P. falciparum**. n: Number of samples, S: Number of polymorphic sites, k: Average number of pairwise nucleotide differences, π: Nucleotide diversity, h: Number of haplotypes, Hd: Haplotype diversity.

| Gene | Locality | n | S | k | π | h | Hd |
|---|---|---|---|---|---|---|---|
| *Pfcrt* | Tobar Donoso | 18 | 2 | 0.418 | 0.028 | 2 | 0.209 |
| | Esmeraldas | 25 | 0 | 0.000 | 0.000 | 1 | 0.000 |
| | San Lorenzo | 5 | 2 | 1.200 | 0.080 | 2 | 0.600 |
| | Colombia | 15 | 3 | 1.090 | 0.080 | 3 | 0.562 |
| *Pfdhps* | Tobar Donoso | 17 | 1 | 0.018 | 0.008 | 2 | 0.118 |
| | Esmeraldas | 24 | 1 | 0.080 | 0.005 | 2 | 0.080 |
| | San Lorenzo | 5 | 0 | 0.000 | 0.000 | 1 | 0.000 |
| | Colombia | 6 | 3 | 1.200 | 0.080 | 3 | 0.600 |
| *Pfdhfr* | Tobar Donoso | 18 | 0 | 0.000 | 0.000 | 1 | 0.000 |
| | Esmeraldas | 27 | 0 | 0.000 | 0.000 | 1 | 0.000 |
| | San Lorenzo | 5 | 2 | 1.200 | 0.080 | 2 | 0.600 |
| | Colombia | 13 | 2 | 0.670 | 0.040 | 3 | 0.410 |
| *Pfmdr-1* | Tobar Donoso | 18 | 0 | 0.000 | 0.000 | 1 | 0.000 |
| | Esmeraldas | 25 | 1 | 0.220 | 0.009 | 2 | 0.220 |
| | San Lorenzo | 5 | 1 | 0.600 | 0.025 | 2 | 0.600 |
| | Colombia | 12 | 1 | 0.530 | 0.022 | 2 | 0.530 |

## Discussion

The efficacy of antimalarials has contributed to the reduction of morbidity and mortality rates of malaria [57]. However, the spread and increase of antimalarial resistance contribute to the emergence of malaria outbreaks and hinder malaria elimination [24,42]. This study focused on the molecular epidemiology of drug resistance genes (*Pfcrt*, *Pfdhps*, *Pfdhfr*, *Pfmdr-1*, *Pfk13* and *Pfaat1*) in *P. falciparum* in northwestern Ecuador between 2019 and 2021. We show *P. falciparum* CQ and pyrimethamine resistance expansion and most importantly the appearance of a mutation associated with proguanil resistance for the first time in Ecuador.

The haplotypes found in *Pfcrt* (Fig. 2a) are clear evidence of strong pressure that CQ has exerted over *P. falciparum* for more than five decades in Ecuador (Fig. 4) [49,58]. It is evident that in Ecuador as in the rest of South America (Cortese et al., 2002b; Sáenz et al., 2015; Valenzuela et al., 2019), CQ resistance has become fixed as all sampled parasites carried the K76**T** coding mutation in *Pfcrt*. Reversal of CQ resistance has not been seen in this region even though CQ is no longer used against *P. falciparum* (Cortese et al., 2002b; Sá et al., 2009). However, in French Guiana, reversal of CQ resistance has been acquired by the C350**R** mutation in *Pfcrt* [60]. The massive administration of CQ to combat malaria in South America since 1950, even as prophylaxis in cooking salt [26,61], caused a strong pressure that resulted in the emergence of CQR parasites [15,62]. Likewise, the indiscriminate use of CQ reported in several areas of the Pacific coast during the 1990s contributed to the selection of CQR parasites [63]. Besides, clonal expansions, very common in South America, may have caused the fast spread and increase of CQR parasites [21], as well as the loss of CQ-sensitive parasites that can compete in absence of CQ pressure.

Other mutations such as H97**Q**, A220**S**, Q271**E**, N326**D**, C350**S**, I356**T/L** and R371**I** in *Pfcrt* have been associated with CQR parasites [16,17]. In this study, H97**Q**, A220**S**, N326**D**, S334**N**, I356**L** and R371**T** were found (Table 1). The presence of these mutant alleles may have contributed to CQ resistance in Ecuadorian *P. falciparum* because it has been suggested that at least three complementary mutations to K76**T** are necessary to confer high degrees of CQ resistance [18].

Resistance to sulfadoxine and pyrimethamine (SP) appears to be more complex than CQR. Resistance mutations in *Pfdhfr* were found in 65% of Ecuadorian samples (Fig. 2c). In contrast, sulfadoxine resistance appears low as only one instance of a mutant *Pfdhps* haplotype, S**G**KAA, (Fig. 2b), was found among the Colombian, but not the Ecuadorian, samples. This difference may stem from the history of drug use in the region. In South America, pyrimethamine was used massively as a prophylactic against malaria in the 1950s [33]. Later, in the 1970s, pyrimethamine was co-administrated with sulfadoxine to combat CQR parasites [14]. Consequently, SP resistance has been reported in South America since 1980 [26]. Specifically, in Ecuador, SP was implemented as a second-line treatment in 1950 and, in 2005, it was used as first-line treatment until 2006 (Fig. 4) [58]. Therefore, the emergence of resistant parasites was caused by the pressure exerted by SP for more than five decades. However, the intermittent use of SP temporarily reduced SP pressure, which may have caused SP-sensitive parasites to continue to circulate in the region. So, the wild-type haplotypes in *Pfdhps* and *Pfdhfr* are still observed in Ecuador as in South

America. Favorably, the intermittent use may have prevented SP resistance from becoming fixed in the region.

Importantly, the genetic monitoring of *Pfdhfr* further suggests that resistance to proguanil has appeared in Ecuador. *P. falciparum* acquires resistance to cycloguanil, the active metabolite of proguanil via combined coding mutations for A16**V** and S108**N/T** in *Pfdhfr* [14,28,64]. In Colombia, A16**V** was detected for the first time in combination with CNCSI and C**I**C**N**I in Nariño between 2012 and 2013 [31]. In this study, A16**V** with CNCSI was identified in two samples from Tobar Donoso (Table 1) and was previously detected in isolates from San Lorenzo (2016) [49]. While A16**V** was reported in Pacific coast parasites, the samples from San Lorenzo and Tobar Donoso are not the same clones. Instead, our IBD analysis shows that A16V was passaged onto a new genomic background through recombination[49]. Atovaquone-proguanil has been commercialized in Nariño, Colombia for many years [31], suggesting that the emergence of A16**V** in Colombia has been caused by the use of atovaquone-proguanil as prophylaxis against malaria [31]. So, the presence of A16**V** in Tobar Donoso could be explained by recent recombination of parasites in the Pacific Coast of Colombia or Ecuador[49]. The possible spread of this mutation in Ecuador and Colombia raises an important point against the future use of proguanil in the region.

At *Pfmdr-1*, a shift in haplotype frequencies across time was found (Fig. 3d). While both common haplotypes have been circulating in South America for many years, the most frequent haplotype in 2019–2021 (the triple mutant NED**F**S**D**F**Y**) has not been sampled previously in Ecuador. Mutations in *Pfmdr-1* have an important role in response to different antimalarials such as CQ, QN, AQ, MQ and LF [6,14,15]. However, South American *P. falciparum* have not shown phenotypic changes in drug response even though Y184**F**, N1042**D** and D1246**Y** are commonly present [20,66]. An *in vitro* assay tested parasites from Nariño with NED**F**S**D**FD and NED**F**S**D**F**Y** haplotypes, which were shown to be sensitive to AQ, MQ and LF [66]. In Esmeraldas, ESM-2013 (isolated from 2013) with Y184**F** and N1042**D** mutation showed sensitivity to QN, LF and AR [20]. Mutations in other genes located in the DV such as *Pfcrt* and *Pfaat1* may therefore influence resistance to these drugs [11,67].

The prevalence of MYRIC haplotype (wild-type) in *Pfk13* (Fig. 2e) shows sensitivity to ARTs in 100% of Ecuadorian *P. falciparum* samples. Previous studies in Ecuador, Colombia, Peru, Venezuela, Brazil, Suriname and French Guiana have not found any mutation in *Pfk13* associated with ART resistance [20,42,43]. However, in Guyana, the C580**Y** mutation was detected in 2010 [41,42]. There is, therefore, concern that C580**Y** could spread to other regions of South America, and molecular studies are key to monitoring for any spread or new emergence. In addition, K189**T** mutation in *Pfk13* was identified in this study and recently reported in Ecuador, Colombia, Venezuela, Brazil, Guyana and French Guiana [42,49]. In Ecuador, AS was used with SP as a first-line treatment in 2006; however, AL was already used as a second-line treatment in 2005 [58]. AL has been the official first-line treatment against *P. falciparum* since 2012 (Fig. 4) [58].

In the last decade, resistance to quinolines such as CQ, QN, AQ and MQ has been associated with mutations in *Pfaat1* [44–46]. The S258**L** mutation identified in this study (Table 1) shows evidence of having been under selection in the Pacific coast of Ecuador and Colombia [49]. Recently, Amambua-Ngwa *et al* (2023) found the same mutation segregating in Gambian parasites, and through CRISPR/Cas9 editing, validated its role in CQ resistance [48]. Likewise, *in vitro* study showed resistance to three compounds not yet cataloged (MMV668399, MMV011895 and MMV007224) when P380**S**, K238**N**, V185**L**, F230**L** mutations and an insertion at codon 35 were present in *Pfaat1* [47]. *Pfaat1* is a highly conserved gene [46], so the emergence of mutations may be related to pressure from quinolines. This pressure could have been caused mostly by CQ and QN, used historically in South America [26,68], and AQ which was introduced in several areas of South America, including the Pacific coast, since 1949 [24,59,69].

As has been observed elsewhere in South America, our measured patterns of resistance haplotype frequency do not track contemporary drug use. Instead, the recent increase in proportions of mutant haplotypes in Ecuador such as CVM**ET** (double mutant) in *Pfcrt*, C**I**C**N**I (double mutant) in *Pfdhfr* and the appearance of NED**F**S**D**F**Y** (triple mutant) in *Pfmdr-1* (Fig. 3) could be explained by clonal expansions caused by parasites from migrant humans and not by the pressure by CQ and SP treatments, because they have not been used against *P. falciparum* in the last two decades. A previous analysis of neutral microsatellites found that outbreaks that occurred in Tobar Donoso and Esmeraldas in 2019 and 2020, respectively, were mostly caused by a single parasite clone [70]. Furthermore, CVM**ET** in *Pfcrt*, C**I**C**N**I in *Pfdhfr* and NED**F**S**D**F**Y** in *Pfmdr-1* were dominant in samples from migrant human hosts in this study (Fig. 2), and they have been found circulating in Nariño since 1999 [24,26,31]. While in northwest Ecuador, CVM**ET** and C**I**C**N**I were reported in low proportions in 2013–2015 [20], and NED**F**S**D**F**Y** was absent, but they are now common in recent samples in this study. This suggests that recent outbreaks that occurred in Ecuador may have resulted from the spread of parasites by migrant humans from Nariño.

Previously, an important human migratory flow that contributes to the spread of parasites on the Pacific coast of Colombia has already been reported [51]. In the present study, the main routes of cross-border and intra-border human migration contributing to the spread of malaria parasites in the North coast of Ecuador and the South coast of Colombia are proposed (Fig. 5). Specifically, the spread of resistant parasites to Ecuador could have occurred in two ways: 1) by Ecuadorians who traveled to Colombia and became infected with parasites from there, and 2) directly by infected Colombians who arrived in Ecuador. The main migration route between Nariño and San Lorenzo for several years has been by their seaports. Therefore, Colombian parasites could have reached the coast of Ecuador by the constant migratory flow along the Pacific coast due to the shellfish trade, legal and illegal mining, agriculture and illegal activities such as drug trafficking through the seaports of Nariño, San Lorenzo and Esmeraldas [71–73]. In addition, it has recently been found that *P. falciparum* populations are shared between Nariño and San Lorenzo [49,50]. Currently, a new road has been opened in this area between Colombia and Ecuador, the bridge over Mataje River that connects Nariño and San Lorenzo [74]. This road will definitely represent an important passage of malaria infected people between Ecuador and Colombia. Finally, the E15 road that connects San Lorenzo and Esmeraldas is an important corridor in the spread of malaria parasites. In addition, the presence of A16**V** in *Pfdhfr* (Table 1) in Tobar Donoso also suggests a process of human migration because this mutation has already found in San Lorenzo [49] and is very common in Nariño [31]. Possibly, the migration of human hosts occurred along of the Mira River, which comes from Nariño, and it is frequently used by Colombians to trade and transport supplies to Tobar Donoso [75]. Additionally, migration by the E10 road, which connects San Lorenzo and Carchi, could also be contributing to the spread of parasites to Tobar Donoso because it is frequently used to reach the south of Carchi province. From there third-order improvised roads and walking trails connect with Tobar Donoso. It is important to note that a helicopter from Esmeraldas transports food, medicine and soldiers to monitor the border at least once a month [75], and could also be considered a route of entry of parasites from Esmeraldas to Tobar Donoso.

Factors such as drug pressure and host human migration influence the emergence and spread of resistant parasites which contribute to the appearance of malaria outbreaks and complicate malaria control and elimination in Ecuador. The findings of this study indicate that chloroquine and pyrimethamine-resistant parasites have increased in recent years, while sulfadoxine and artemisinin-sensitive parasites prevail in Ecuador. Therefore, the current treatment (artemether + lumefantrine) is still effective against *P. falciparum*. In addition, the genotypes of *P. falciparum* show rapid fluctuations within the region, likely due to the boom-bust dynamics of clonal lineages coupled with frequent migration of human hosts across the Ecuador-Colombia border. Finally, this study shows the importance of the epidemiological characterization of resistance in *P. falciparum* in a region in the process of eliminating malaria: genetic surveillance of drug resistance genes is key to respond in a timely manner in case of therapeutic failure and prevent the spread of malaria.

### Methods

## Ethics statement

The study was approved by the Research Ethics Committee on Human Beings of Pontificia Universidad Católica del Ecuador registered in the document CEISH-571-2018 and by the Ministry of Public Health (MPH) of Ecuador registered in the document MSP-DIS-2019-044-O.

## Study area and parasite population

The study was carried out in Ecuador (South America) from 2019 to 2021, with samples collected in Esmeraldas and Carchi provinces located in the Northwest on the border with Colombia (Fig. 1). Sixty-seven blood samples were collected on Whatman filter paper from patients diagnosed with *P. falciparum* by microscopy or RDTs (Rapid Diagnostic Tests). Samples were collected by personnel of the MPH between 2019 and 2021 and delivered to CISeAL (Centro de Investigación para la Salud en América Latina) for molecular analysis. During this period, 702 infections by *P. falciparum* were registered in Northwest Ecuador [4]. Samples of local Ecuadorian cases corresponded to the counties of Esmeraldas

(40.29%), San Lorenzo (7.46%) and Tobar Donoso (26.86%). The remaining 25.37% corresponded to migrants from Nariño, Colombia diagnosed in San Lorenzo. Additionally, data of *P. falciparum* samples (2013–2015 period) from a previous study in Ecuador were included for temporal comparisons [20].

## DNA extraction

DNA extraction of *P. falciparum* was performed from blood samples on Whatman filter paper stored at 4°C, using the commercial Purelink™ Genomic DNA Mini Kit, INVITROGEN following the protocol recommended by the manufacturer (Invitrogen, Life Technology, USA), and stored at -20°C for its molecular analysis.

## Amplification of drug resistance genes

DNA was amplified for molecular markers of drug resistance genes: *Pfcrt* (codons 72–76), *Pfdhps* (codons 436, 437, 540, 581 and 613), *Pfdhfr* (codons 50, 51, 59, 108 and 164), *Pfmdr-1* (codons 86, 130, 144, 184, 1034, 1042, 1226 and 1246) and *Pfk13* (codons 476, 493, 539, 543 and 580).

Polymerase Chain Reaction (PCR) solution contained 12.5 µl of Colorless GoTaq Master Mix (Promega, Madison, WI, USA), 2.5 µl of $MgCl_2$ (25 mM) (Promega, Madison, WI, USA), 3 µl of ultrapure $H_2O$, 1 µl of primers forward and reverse (10 µM) and 5 µl of DNA. Nested PCR protocols (Table 3) established in previous studies were used [20]. Amplifications were carried out in SureCycler 8800 (Agilent Technologies, USA).

Amplicon sizes were verified in 2% agarose gel and observed in UVP BioDoc-It digital transilluminator (UVP, CA, UK). After confirmation, amplified DNA was sent for purification and Sanger sequencing to Macrogen, South Korea.

Table 3

**Primers used for the nested PCR for molecular markers of drug resistance genes in P. falciparum**. *Pfcrt*: *Plasmodium falciparum* chloroquine resistance transporter; *Pfdhfr*: *Plasmodium falciparum* dihydropteroate synthetase; *Pfdhps*: *Plasmodium falciparum* dihydrofolate reductase; *Pfmdr-1*: *Plasmodium falciparum* multidrug resistance 1; *Pfk13*: *Plasmodium falciparum* kelch 13.

| Gen | Primers 5' – 3' | Amplicon | Reference |
|---|---|---|---|
| *Pfcrt* | **F1**: AGCAAAAATGACGAGCGTTATAG | 559 pb | Griffing *et al.*, 2010 |
| | **R1**: ATTGGTAGGTGGAATAGATTCTC | | |
| | **F2**: TTTTTCCCTTGTCGACCTTAAC | 264 pb | |
| | **R2**: AGGAATAAACAATAAAGAACATA ATCATAC | | |
| *Pfdhfr* | **F1**: TCCTTTTTATGATGGAACAAG | 633 pb | Alam *et al.*, 2011 |
| | **R1**: AGTATATACATCGCTAACAGA | | |
| | **F2**: TTTATGATGGAACAAGTCTGC | 616 pb | |
| | **R2**: ACTCATTTTCATTTATTTCTGG | | |
| *Pfdhps* | **F2**: GATTCTTTTTCAGATGGAAA | 770 pb | Fierro, 2017 |
| | **R2**: TTCCTCATGTAATTCATCTGA | | |
| | **F1**: AACCTAAACGTGCTGTTCAA | 711 pb | |
| | **R1**: AATTGTGTGATTTGTCCACAA | | |
| *Pfmdr-1* (86) | **F1**: CCGTTTAAATGTTTACCTGCAC | 1014 pb | Vinayak *et al.*, 2010 |
| | **R1**: TGGGGTATTGATTCGTTGCAC | | |
| | **F2**: GTATGTGCTGTATTATCAGGAG | 746 pb | |
| | **R2**: AGCCTCTTCTATAATGGACATG | | |
| *Pfmdr-1* (1034) | **F1**: TTTAAAGATCCAAGTTTTTTAAT ACAGGAAGC | 924 pb | Montenegro *et al.*, 2021 |
| | **R1**: CTTACTAACACGTTTAACATCTTC CAATG | | |
| | **F2**: AAGCTATTGATTATAAAAATAAAG GACAAAAAAGAAG | 761 pb | |
| | **R2**: GGACATATTAAATAACATGGGTT CTTGAC | | |

| Gen | Primers 5' – 3' | Amplicon | Reference |
|---|---|---|---|
| *Pfk13* | **F1**: GCAAATAGTATCTCGAAT | 2126 pb | Talundzic *et al*., 2015 |
| | **R1**: CTGGGAACTAATAAAGAT | | |
| | **F2**: GATAAACAAGGAAGAATATTCT | 748 pb | |
| | **R2**: CGGAATCTAATATGTTATGTTCA | | |

## Determination of genotypes in drug resistance genes

The NF54 strain of *P. falciparum* was used as a control to identify SNPs (Single Nucleotide Polymorphisms) in *Pfcrt* (codons 72–76), *Pfdhps* (codons 436, 437, 540, 581 and 613), *Pfdhfr* (codons 50, 51, 59, 108 and 164), *Pfmdr-1* (codons 86, 130, 144, 184, 1034, 1042, 1226 and 1246) and *Pfk13* (codons 476, 493, 539, 543 and 580). All sequences were analyzed and aligned in Geneious software version 2020.0.1 (Biomatters, Inc, Newark, NJ, USA).

## Whole-Genome Sequencing

Fifty-four DNA samples underwent selective whole genome amplification (SWGA) to boost representation of *P. falciparum* DNA within total DNA extracts [55]. The Broad Institute Genomics Platform then constructed NEBNext libraries and performed 150-bp paired-end sequencing on an Illumina NovaSeqSP instrument. Sequences were aligned and variants called using a standard BWA-GATK3 pipeline following the best practices established by the Pf3k consortium [76]. The WGS results were used to confirm the genotypes obtained by Sanger sequencing and to identify variants in the same genes outside the Sanger-sequenced regions.

## Data Analysis

GraphPad Prism version 8.0.2 was used to calculate frequencies and make comparative graphs of *Pfcrt*, *Pfdhps*, *Pfdhfr*, *Pfmdr-1* and *Pfk13* haplotypes in each locality from the 2013–2015 and 2019–2022 periods. To compare the frequencies of haplotypes between two different time points, a Chi-square ($x^2$) test was used. Significance was defined as $p < 0.05$. The number of polymorphic sites (S), the average number of pairwise nucleotide differences (k), nucleotide diversity (π), number of haplotypes (h) and haplotype diversity (Hd) were calculated in DnaSP software version 6.12.03.

## Relatedness Analysis

We estimated relatedness between all genome pairs using the program hmmIBD [77]. The algorithm estimates fractional (genome-wide) relatedness as identity by descent (IBD) and provides IBD status at all genomic coordinates included in the analysis. We used the length and position of these IBD tracts to infer relatedness at the *Pfdhfr* locus. The IBD analysis included both the Ecuador samples from this study and 219 additional samples from the South American Pacific Coast originally analyzed in Carrasquilla et al (2022)[49]. We excluded from the IBD analysis any genome with less than 30% of the genome covered at 5x.

## Declarations

#### Acknowledgements

We thank to the personnel of the Ministry of Public Health of Ecuador for providing the samples for this study and all patients who participated.

#### Author contributions

IÑ performed the experiments; IÑ and FES designed the study. IÑ, FES, AME and DEN wrote the manuscript; AME and DEN obtained WGS data.

#### Data availability statement

Data analyzed in this study is available in CISeAL and can be requested to Dr. Fabián Sáenz. All whole-genome sequence data are available on the NCBI Sequence Read Archive as BioProject PRJNA759192.

#### Competing interests statement

The authors declare that they have no competing interests.

#### Ethics approval and consent to participate

The study was approved by the Research Ethics Committee on Human Beings of Pontificia Universidad Católica del Ecuador (approvals #: CEISH-571-2018) and the Ministry of Public Health of Ecuador (approvals #: MSP-DIS-2019-044-O).

#### Funding

This study was funded by Pontificia Universidad Católica del Ecuador (grant #QINV0084-IINV529010100 and Fondo Publícalo). This project has been funded in whole or in part with Federal funds from the National Institute of Allergy and Infectious Diseases (NIAID), National Institutes of Health, Department of Health and Human Services, under Grant Number U19AI110818 to the Broad Institute awarded to DEN.

## Figures

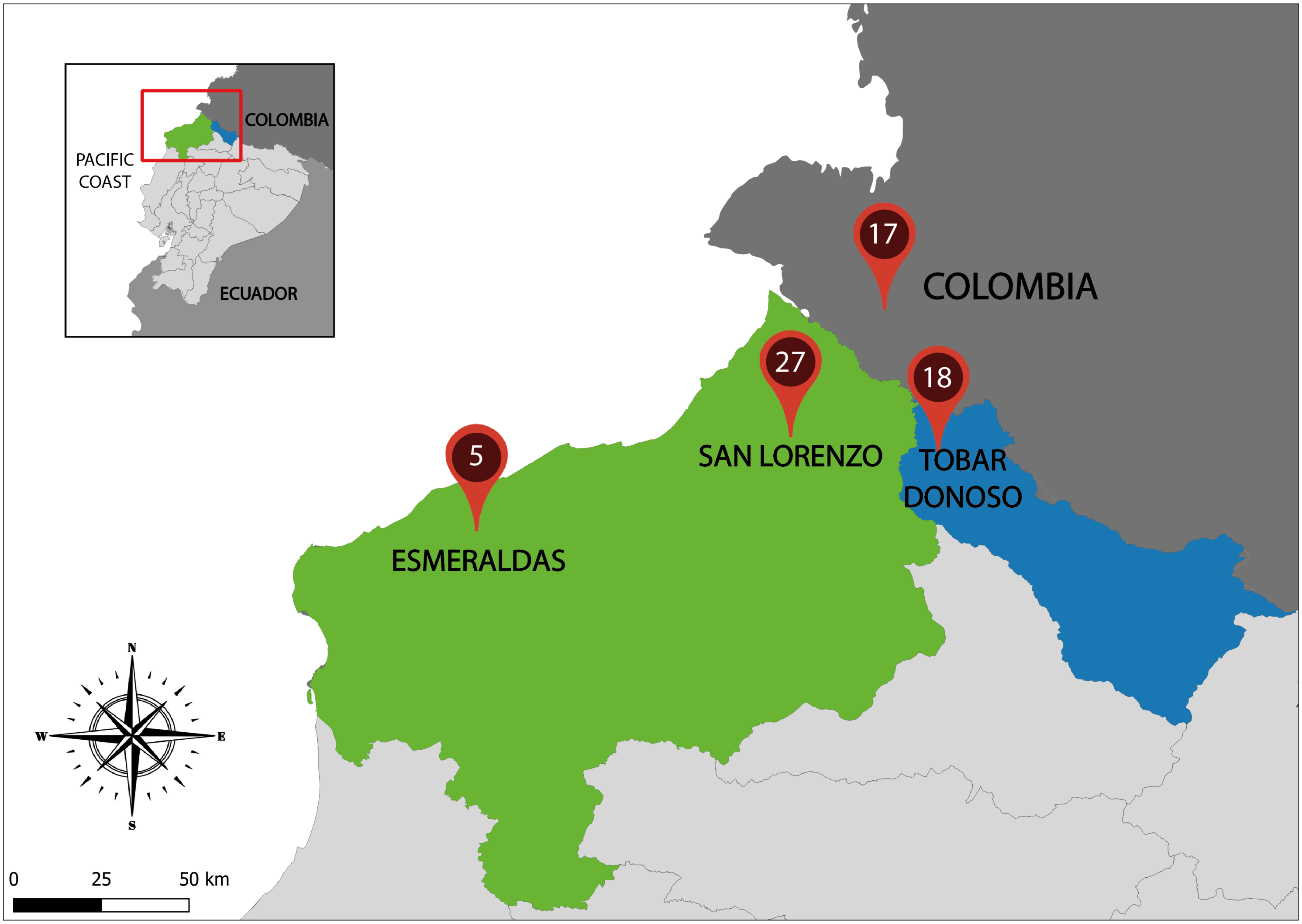

### Figure 1

**Study sites.** Samples collected from patients in northwest Ecuador: Esmeraldas (n=27), San Lorenzo (n=5), Tobar Donoso (n=18) and Colombia (n=17).

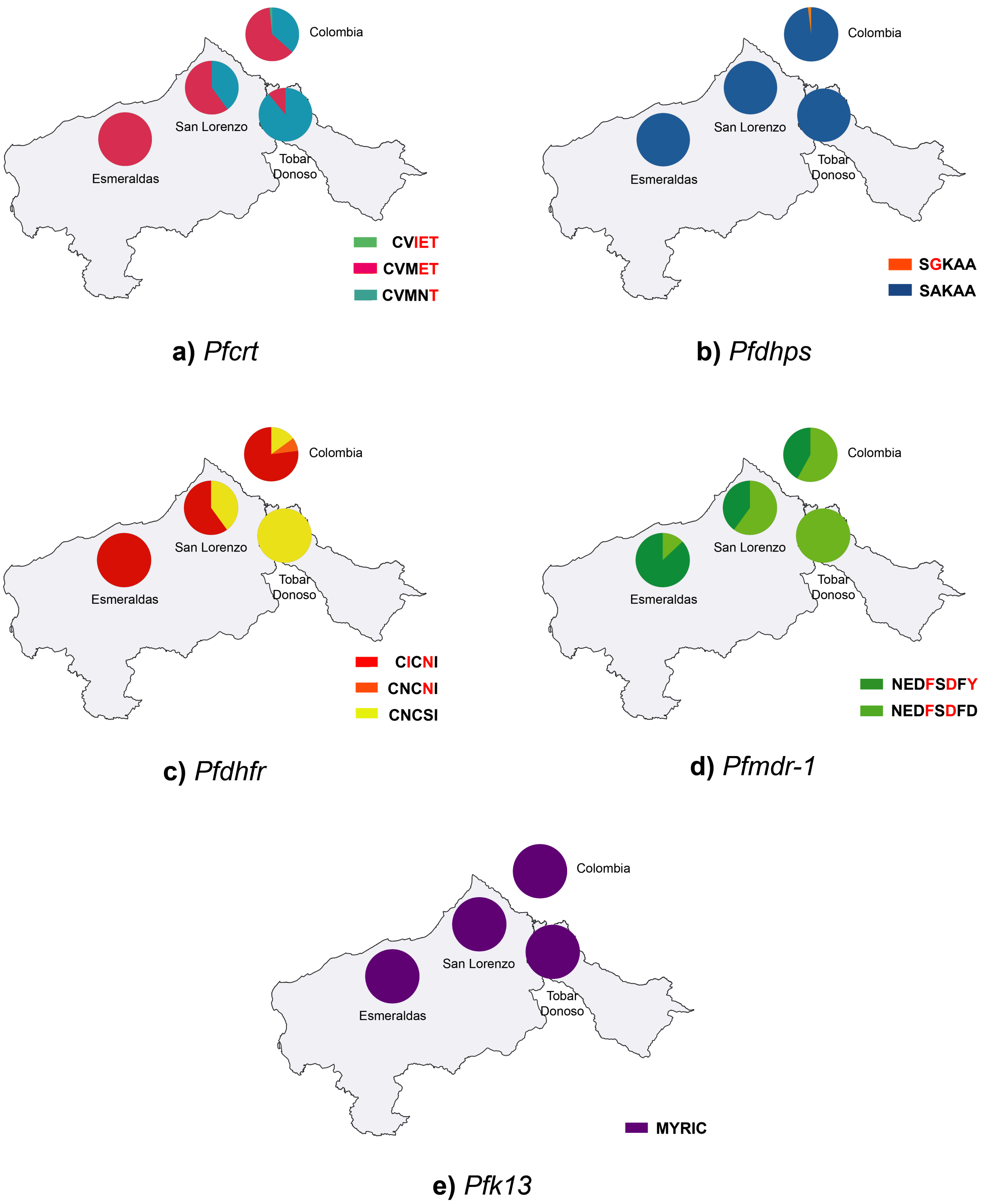

**Figure 2**

**Geographic distribution of frequencies of drug resistance haplotypes in the 2019-2022 period.** a. Haplotypes of *Pfcrt*(72-76), b. Haplotypes of *Pfdhps* (436, 437, 549, 581 and 613), c. Haplotypes of *Pfdhfr* (50, 51, 59, 108 and 164), d. Haplotypes of *Pfmdr-1*(codons 86, 130, 144, 184, 1034, 1042, 1226 and 1246), e. Haplotypes of *Pfk13* (codons 476, 493, 539, 543 and 580).

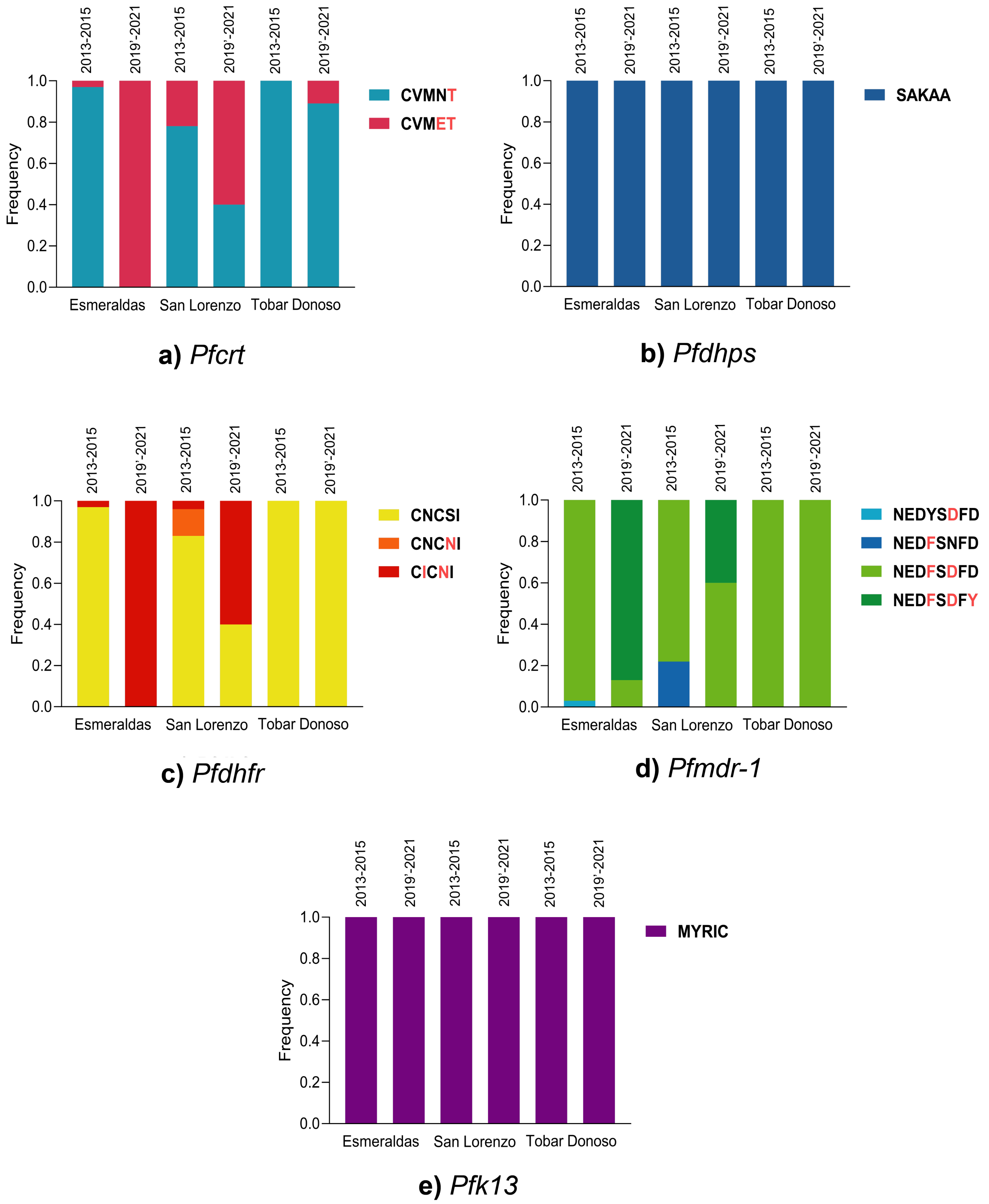

### Figure 3

**Comparative frequencies of drug resistance haplotypes in Northwest Ecuador between the 2013-2015 and 2019-2022 periods.** a. Haplotypes of *Pfcrt* (72-76), b. Haplotypes of *Pfdhps* (436, 437, 549, 581 and 613), c. Haplotypes of *Pfdhfr* (50, 51, 59, 108 and 164), d. Haplotypes of *Pfmdr-1* (codons 86, 130, 144, 184, 1034, 1042, 1226 and 1246), e. Haplotypes of *Pfk13* (codons 476, 493, 539, 543 and 580).

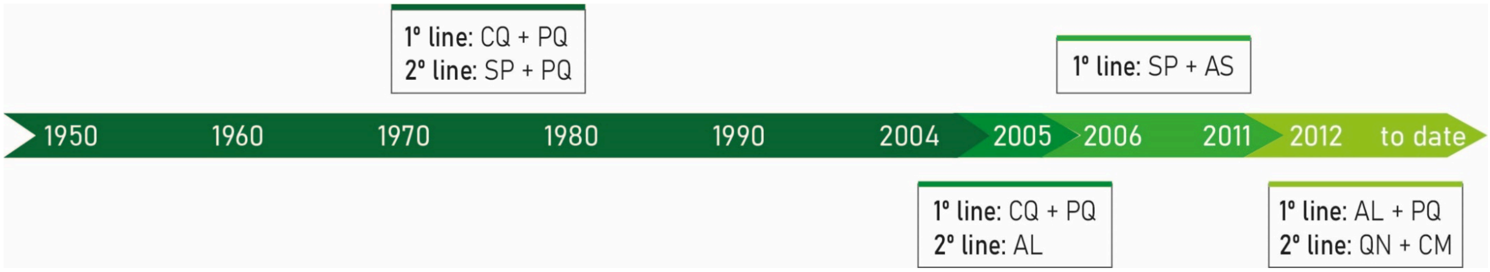

### Figure 4

**Antimalarial treatments against *P. falciparum* in Ecuador**. Timeline of antimalarial treatments since 1950 according to the Ministry of Public Health of Ecuador. Chloroquine (CQ), primaquine (PQ), sulfadoxine-pyrimethamine (SP), artemether-lumefantrine (AL), artesunate (AS), quinine (QN) and clindamycin (CM). Source: Aguilar, 2018

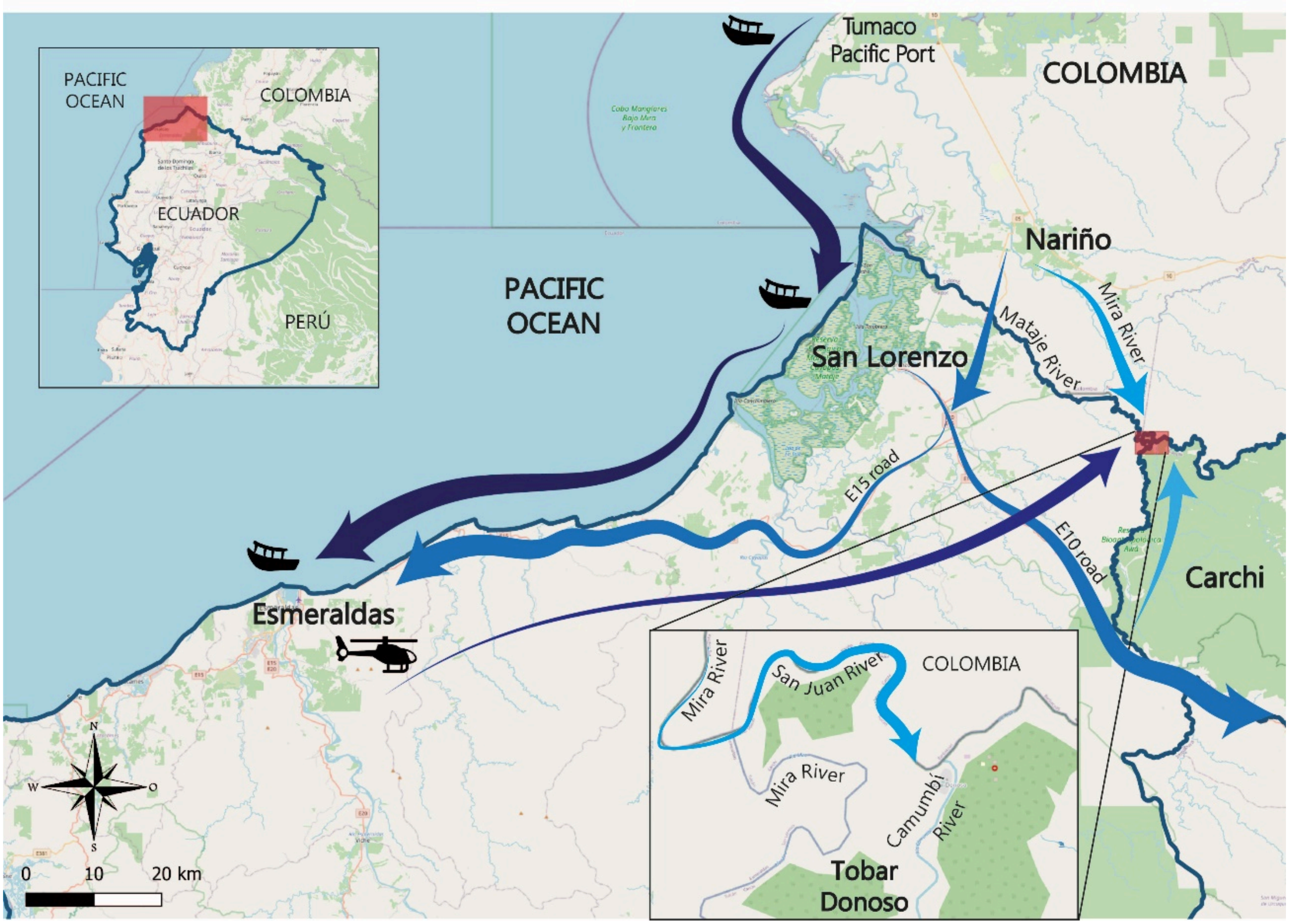

### Figure 5

**Cross-border and intra-border routes of entry of *P. falciparum* in northwestern Ecuador.** Possible routes of the human migration used for the passage of *P. falciparum* in the south coast of Colombia and north coast of Ecuador. Elaborated according to the information collected from oral reports.
